# The role of collective motion in the ultrafast charge transfer in van der Waals heterostructures

**DOI:** 10.1038/ncomms11504

**Published:** 2016-05-10

**Authors:** Han Wang, Junhyeok Bang, Yiyang Sun, Liangbo Liang, Damien West, Vincent Meunier, Shengbai Zhang

**Affiliations:** 1 Department of Physics, Applied Physics, and Astronomy, Rensselaer Polytechnic Institute, Troy, New York 12180, USA; 2 Spin Engineering Physics Team, Korea Basic Science Institute (KBSI), Daejeon 305-806, Korea; 3 Center for Nanophase Materials Sciences, Oak Ridge National Laboratory, Oak Ridge, Tennessee 37831, USA

## Abstract

The success of van der Waals heterostructures made of graphene, metal dichalcogenides and other layered materials, hinges on the understanding of charge transfer across the interface as the foundation for new device concepts and applications. In contrast to conventional heterostructures, where a strong interfacial coupling is essential to charge transfer, recent experimental findings indicate that van der Waals heterostructues can exhibit ultrafast charge transfer despite the weak binding of these heterostructures. Here we find, using time-dependent density functional theory molecular dynamics, that the collective motion of excitons at the interface leads to plasma oscillations associated with optical excitation. By constructing a simple model of the van der Waals heterostructure, we show that there exists an unexpected criticality of the oscillations, yielding rapid charge transfer across the interface. Application to the MoS_2_/WS_2_ heterostructure yields good agreement with experiments, indicating near complete charge transfer within a timescale of 100 fs.

## Abstract (web summary)

Ultrafast charge transfer has been observed experimentally in van der Waals heterostructures. Here, Wang *et al*. study theoretically the collective exciton motion at the interface. They show that the oscillations lead to charge transfer, with a rate that depends critically on the coupling and on the layer stacking.

## Graphical abstract

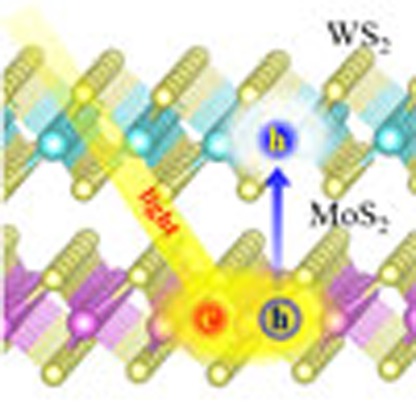

Charge transfer is a fundamental process governing numerous biological, chemical and physical systems. Electrons or holes transferred to different regions or functional groups can trigger chemical reactions and are integral to phenomena such as photosynthesis and water splitting^1^,^2^. In heterostuctures, which are a basic platform for solid-state applications such as solar cells, light-emitting diodes and high-mobility transistors^3^,^4^,^5^, charge transfer controls electron–hole recombination and hence plays a crucial role in device operation^6^,^7^. While the structure of traditional (covalent) heterostructures are limited by the stringent requirement of lattice matching between constituent materials to avoid defect formation and large strain at the interface^8^, the severity of such issues are greatly diminished in the emerging van der Waals (vdW) heterostructures consisting of two-dimensional atomic crystals^9^,^10^,^11^. The vdW heterostructures are very promising as their components can be chosen from a variety of systems, such as graphene, hexagonal boron nitride, transition metal dichalcogenides (TMDs), phosphorene and silicene. These atomically thin materials present a set of attractive physical properties such as high thermal and electrical conductivity^12^, Klein tunnelling^13^, valleytronics^14^,^15^,^16^, strong absorption of light^17^ and strong photoluminescence^17^,^18^,^19^. For vdW interfaces, one may expect that the component materials are relatively isolated from each other maintaining their individual properties because the interfacial vdW spacing is considerably wider than a covalent or ionic chemical bond, and hence electronic coupling between the layers is expected to be weak. This presumed weak coupling has motivated recent proposals for using such materials for photovoltaic and diode-like current rectification applications^20^,^21^,^22^,^23^. This makes the recent findings of photoluminescence and femtosecond pump-probe spectroscopy experiments^20^,^24^ that the charge transfer in MoS_2_/WS_2_ is an ultrafast phenomena (within 100 fs) quite surprising. Similarly, fast charge transfer has also been observed for the MoS_2_/MoSe_2_ heterostructure^22^, suggesting that in these new heterostructures, the physics of charge transfer may fundamentally depart from traditional pictures of non-coherent charge transfer^25^,^26^,^27^. Developing a basic understanding of the mechanisms of charge transfer across the vdW hetostructure is not only of fundamental interest but essential for the successful application of these materials.

In this work, time-dependent density functional theory (TDDFT) is combined with molecular dynamics (MD) to understand the physics governing the ultrafast hole transfer from MoS_2_ to WS_2_. We find that significant hole transfer takes place within a timescale of 100 fs of the initial excitation in agreement with experiments^20^,^24^. Although conventional wisdom suggests that throughout this process accumulated holes would lead to a dipole that would tend to hamper further hole transfer—our analysis of quantum states dynamics during the transfer process suggests precisely the opposite. Our study reveals a strong dynamic coupling, whereby charge transfer across the heterostructure significantly increases the coupling between the hole states in MoS_2_ and WS_2_. It follows that after an initial induction time of relatively slow charge transfer, the charged dipole layer causes the rapid transfer of holes across the heterostructure despite the fact that the internal electric field opposes the direction of hole transfer. A model for the hole transfer shows that this mechanism is critically dependent on the magnitude of the dipole transition matrix element, which determines the coupling across the heterostructure. As a result, the existence of ultrafast charge transfer is sensitive to the atomistic details of the interface, in particular the heterostructure stacking. While the energetically favourable 2H stacking (in which the S atoms of WS_2_ lie directly above Mo) is found to exhibit ultrafast charge transfer, dipole coupling in 3R and AB′ stacking configurations^28^,^29^ (in which the W atom of WS_2_ lies directly above S or Mo from MoS_2_, respectively) is found to remain below the minimum threshold, resulting in much slower charge transfer.

## Results

### Real-time TDDFT-MD

**Figure 1:**
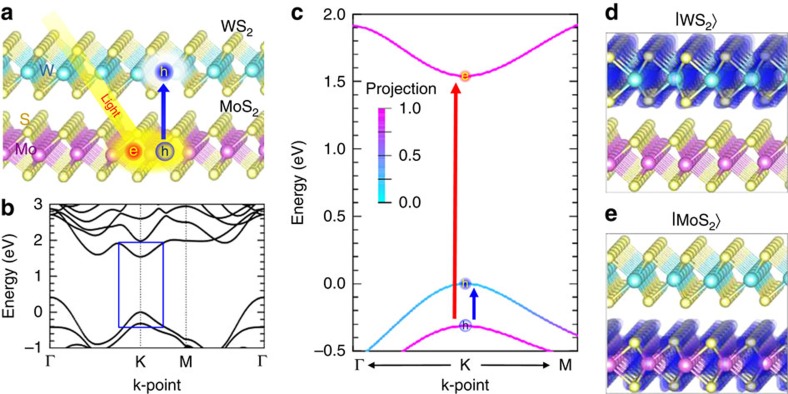
Atomic and electronic structures of MoS_2_/WS_2_ heterostructures. (**a**) Illustration of the heterostructure, where a WS_2_ monolayer lies on top of a MoS_2_ monolayer. Electron and hole carriers excited by incident light separate by hole transfer into the WS_2_. (**b**) Band structure of the heterostructure, showing an indirect bandgap from Γ to K. The smallest direct transition with appreciable absorption is from the (VBM-1) state to the CBM in the vicinity of K. (**c**) Band structure in the vicinity of K-point in the Brillouin zone. From the K-point at the centre, right and left are directions toward Γ and M points, respectively. The incident light excites electron in |MoS_2_〉 to the conduction band minimum, and hole is transferred into |WS_2_〉. The energy of the valence band maximum at the K-point is set to zero. Colour indicates the degree of projection of each state onto the MoS_2_ layer. A projection of one (pink) indicates complete localization of the state to the MoS_2_ layer and that of zero (blue) indicates localization to the WS_2_ layer. (**d**,**e**) Isosurface plots of two valence band charge density at the K-point. The local VBM, which we denote |WS_2_〉, presents almost entirely localized charge to WS_2_ (**d**), but the (VBM-1) state, which we denote|MoS_2_〉, is almost entirely localized to MoS_2_ (**e**).

To investigate the photo-induced charge transfer dynamics at the MoS_2_/WS_2_ interface, we built a structural model and setup similar to the experimental situation^20^,^24^, see Fig. 1a, to choose an initial occupation of electrons associated with the optical excitation, and simultaneously iterate the combined electron and ionic dynamics using time domain TDDFT-MD (see the Methods section). The appropriate initial excitation is determined through examination of the band structure shown in Fig. 1b. We find that the gap is indirect with the valence band maximum (VBM), in which the wave function is delocalized to both WS_2_ and MoS_2_, at Γ and the conduction band minimum (CBM), in which the wave function is localized to MoS_2_, at the K-point of the Brillouin zone, consistent with previous findings^17^,^30^. In the vicinity of the K-point, shown in the blue rectangle and enlarged in Fig. 1c, a type-II alignment develops where the local VBM, which we denote |WS_2_〉 presents a charge density almost entirely localized to WS_2_. Conversely, the state immediately below the VBM, the (VBM-1) state, is almost entirely localized to MoS_2_ and is denoted by |MoS_2_〉 in Fig. 1c. As optical absorption via the indirect transition (VBM→CBM) or involving charge transfer across the interface (VBM(at K)→CBM) is negligible^24^,^30^, the predominate optical absorption is associated with the direct excitation of electrons from the (VBM-1) state to the CBM in the vicinity of K, as indicated by the red arrow in Fig. 1c. The subsequent hole transfer observed in the experiment^20^,^24^ results from the type-II alignment, whereby the holes can substantially reduce their energy by transferring from |MoS_2_〉 to |WS_2_〉, as indicated by the blue arrow in Fig. 1c. Hence, in the TDDFT-MD simulation, two electrons were excited from the |MoS_2_〉 state to the CBM and our subsequent analysis focuses on the hole dynamics.

**Figure 2:**
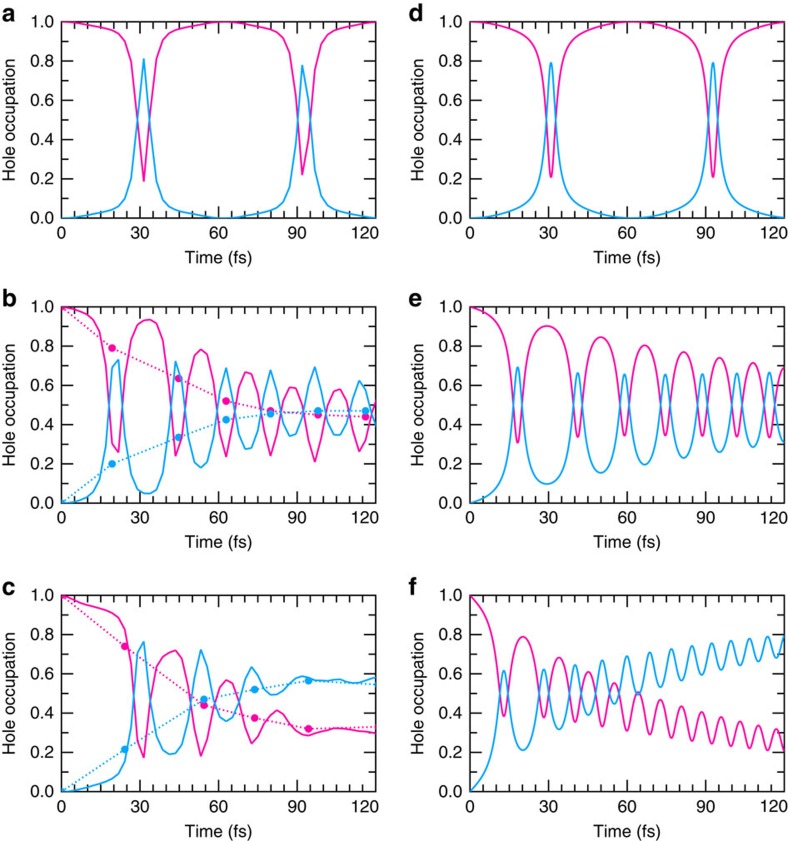
Time evolution of the hole occupation. The hole occupations *ρ*_h_(MoS_2_) and *ρ*_h_(WS_2_) are shown in pink and blue, respectively, as a function of time for the three separate TDDFT-MD simulations with 2H stacking: (**a**) the clamped ion case (**b**) mobile ions at a temperature of 77 K and (**c**) mobile ions at a temperature of 300 K. For the clamped ion case (**a**), we see large periodic oscillations in the hole occupation which are not associated with the ionic dynamics of the system. For the finite temperature cases (**b**,**c**), the average occupations of the hole for each period are indicated by a set of points which are connected by a dashed line as a guide to the eye. As time progresses, the average hole occupation of |WS_2_〉 increases and the average hole occupation of |MoS_2_〉 decreases, indicating charge transfer via dissipation through electron-phonon coupling. (**d**) The dynamics of the hole occupation of the model Hamiltonian presented in equation (2), where the parameters used are indicated in Table 1. The model well produces the oscillations seen in the fixed ion case (**a**). By fitting the coupling of the model to a heat bath containing a receiving mode at 400 cm^−1^, the qualitative features of the hole dynamics for both the 77 and 300 K can be qualitatively reproduced as shown in **e**,**f**, respectively.

**Figure 3:**
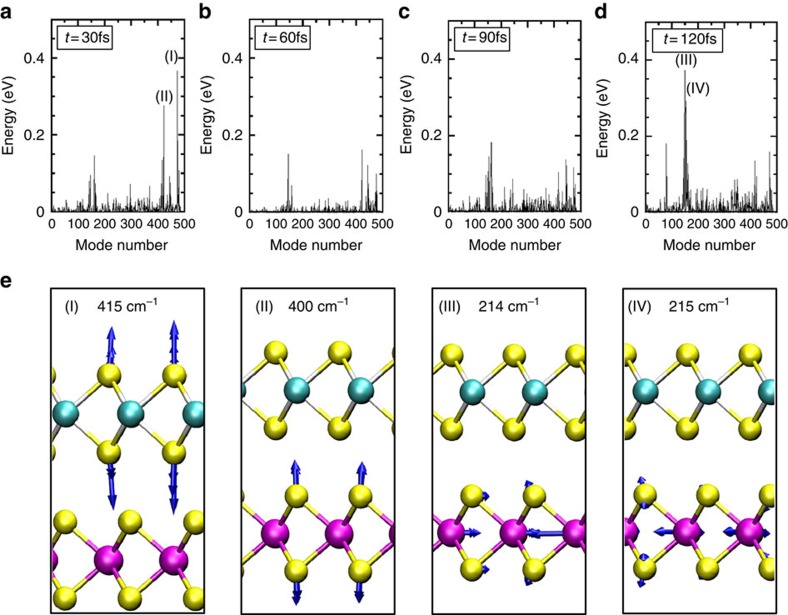
Energy of normal modes of the system as a function of time. This depicts the increase in energy of every vibrational mode of the system as a function of time throughout the TDDFT-MD dynamics of the 77 K simulation. The total energy of each normal mode is indicated at 30, 60, 90 and 120 fs in panel (**a**–**d**), respectively. The four normal modes which pick up the largest amount of energy throughout the simulation are labelled (I), (II), (III) and (IV) with mode numbers of 420, 471, 149 and 153 and corresponding frequencies 415, 400, 214 and 215 cm^−1^, respectively. The displacement eigenvectors associated with these normal modes are depicted by the blue arrows shown in **e**. On the basis of the frequencies and vibrational motion shown in **e**, out-of-plane oscillations of S atoms for (I) and (II), these two phonon modes clearly correspond to the *A*_1*g*_ Raman mode of the MoS_2_ layer and *A*_1*g*_ Raman mode of the WS_2_ layer, respectively. Note that the *A*_1*g*_ mode is one of the most prominent Raman modes in TMDs^32^,^45^, indicating strong electron-phonon coupling for *A*_1*g*_ phonons. Similarly, the modes labelled (III) and (IV) correspond to longitudinal acoustic (LA) phonons with non-zero wavevector. Notably, phonons from the LA branch at the M point have been found to exhibit appreciable signals in the resonant Raman scattering, also suggesting strong electron-phonon coupling for LA phonons^31^.

Figure 2a–c shows the time evolution of the hole (h) occupation *ρ*_h_, calculated by projecting the time-evolved hole wave function *ϕ*(*t*) onto |MoS_2_〉 and |WS_2_〉, that is, *ρ*_h_(MoS_2_)=|〈MoS_2_|*ϕ*(*t*)〉|^2^ and *ρ*_h_(WS_2_)=|〈WS_2_|*ϕ*(*t*)〉|^2^, for the case of 2H stacking at the three different ionic temperatures, *T*=0, 77 and 300 K, respectively. Let us first consider the case of 77 K. Figure 2b shows that *ρ*_h_ oscillates by periodically filling and emptying |MoS_2_〉 and |WS_2_〉 states at a period of approximately 30 fs, albeit with a damped amplitude. The decay of the *ρ*_h_(MoS_2_) is significant, indicating that the average hole position is transitioning in time, from being primarily localized on |MoS_2_〉 to reside on either side of the interface, with nearly equal probability. As the energy of the hole is lower in |WS_2_〉 than in |MoS_2_〉, as shown in Fig. 1c, this change in expectation value for the hole position is associated with an energy relaxation process—wherein excitation energy of the hole is being dissipated by phonons throughout the transition. The same effect, although even more pronounced, is also observed at 300 K, as shown in Fig. 2c. To investigate which phonons are associated with the decay process, we determine the energy of each normal mode of the system as a function of time, as shown for several time snapshots throughout the 77 K simulation in Fig. 3a–d. Here it can be seen that the *A*_1*g*_ Raman active modes and phonons associated with the longitudinal acoustic branch with non-zero wavevectors pick up substantial energy in the decay process. In particular, the strong coupling of the two *A*_1*g*_ phonon modes, involving out-of-plane vibrations of S atoms shown in Fig. 3e, is due to the large associated modification of the distance between the vdW layers. It follows that these modes present a large response to a transverse electric field associated with charge transfer as evidenced by the substantial peaks of the *A*_1*g*_ modes in the Raman spectra of MoS_2_ and WS_2_ (refs 31, 32). While the longitudinal acoustic phonons also show a marked increase in energy at approximately 120 fs, we note that this may be due to subsequent phonon–phonon relaxation of the higher energy *A*_1*g*_ modes.

To better understand the hole dynamics and the role of phonons in the charge transfer process, we decouple the ionic motion from the hole dynamics by performing a TDDFT simulation where the ionic positions are frozen throughout the simulation, as shown in Fig. 2a. The large charge sloshing and the lack of amplitude dampening here are further evidence that it is indeed the electron–phonon coupling that is responsible for the dissipation. In addition, it confirms that the observed hole oscillation is not the result of ionic motion. Interestingly, the oscillation is not sinusoidal as one would expect from a superposition of the two states, |MoS_2_〉 and |WS_2_〉, but rather it can be characterized by broad mesas with sharp peaks. This indicates that the coupling between |MoS_2_〉 and |WS_2_〉, which governs the characteristic frequency of oscillation, gains time dependence throughout the simulation; initially being quite small and becoming more substantial near the peaks. Furthermore this dynamic coupling is purely an electronic phenomenon and is not associated with the ionic vibration, since these were kept fixed throughout this simulation. As such, the mechanism for coherent charge oscillations here are distinct from those proposed for organic photovoltaic blend^33^ and artificial light-harvesting system^34^, wherein the charge oscillations are associated with the frequency of a vibrational mode of the system. To understand the mechanism giving rise to the nonlinear charge oscillations, time-dependent coupling and ultimately the ultrafast charge transfer in the vdW heterostructure, we now further examine the electron dynamics of the system.

### Mechanism for ultrafast coherent charge oscillations

**Figure 4:**
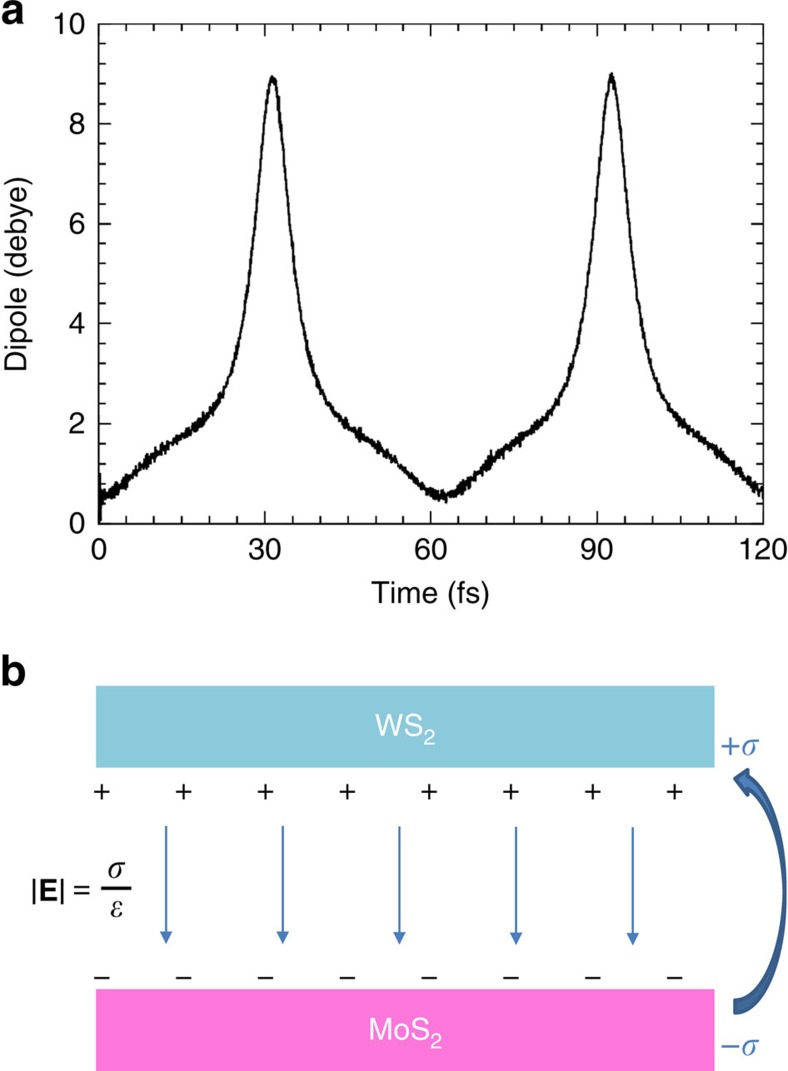
Induced transverse electric field. (**a**) The difference in the total dipole calculated throughout the TDDFT-MD simulation of the hole dynamics with fixed ionic positions and the dipole of the ground-state heterostructure, as a function of time. The sharp peaks in the dipole moment correspond to peaks in the hole occupation on WS_2_ seen in Fig. 2a,b Schematic illustration of a simple parallel plate capacitor view of the heterostructure. Excess charge density *σ* caused by the charge transfer induces electric field 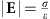 between the plates.

Much insight can be gained on the mechanism for the coherent charge oscillations by considering the excitation-induced interfacial dipole and its time evolution, as shown in Fig. 4a. While the origin of this oscillating interfacial dipole is hole transfer from |MoS_2_〉 to |WS_2_〉, here it should be noted the importance of the collective behaviour of the holes during transport. To mimic the coherent excitation associated with a pump laser setup,^20^ the initial conditions of the TDDFT-MD simulation contain two holes with identical characteristics. Throughout the course of simulation, they maintain identical characteristics with both single-particle hole wavefunctions evolving identically in time. Furthermore, the periodic boundary conditions represent an entire sheet of charges, and as a result charge transfer from MoS_2_ to WS_2_ is associated with the collective motion of holes across the interface with a resulting macroscopic dipole similar to the parallel plate capacitor schematically shown in Fig. 4b. As this macroscopic dipole is responsible for modulating the hole dynamics, tests indicate that for slightly different initial hole character, the results do not alter qualitatively. While the **E**-field produced in this manner opposes hole transfer from MoS_2_ to WS_2_, it significantly enhances the coupling between the two states and hence alters the characteristic timescale of the oscillations.

This phenomenon can be understood in the context of an elementary model where a single hole exists in a two-state system (either in MoS_2_ or WS_2_) in the presence of an external electric field representing the hole's interaction with the dipole generated by the other holes in the system. Assuming the geometry shown in Fig. 4b, the Hamiltonian associated with the uniform **E**-field in the z-direction, perpendicular to the interface,


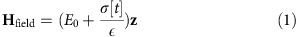


where *E*_0_ is the initial magnitude of the z-component of the electric field associated with charge rearrangement due to the optical excitation and 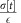 is the time-dependent field, which is dynamically generated due to hole transport from MoS_2_ to WS_2_. Using the ground-state eigenfunctions |WS_2_〉 and |MoS〉 as basis and choosing the origin between the two slabs, the Hamiltonian of the two-state system in the external field becomes,


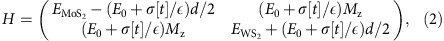


where 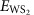 and 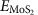 are the hole energies of the ground-state eigenfunctions of the heterostructure, *d* is the effective distance between the two layered materials, seen here as two plates of the capacitor, *d*=(〈WS_2_|*z*|WS_2_〉−〈MoS_2_|*z*|MoS_2_〉), and *M*_z_ is the magnitude of the z-component of the dipole transition matrix element between the heterostructures, 〈WS_2_|*z*|MoS_2_〉. Here it can be seen that the electric field contributes to both the diagonal and off-diagonal matrix elements. While the diagonal ones reduce the energy difference between the two levels that drive hole transfer, the off-diagonal elements increase the dipole coupling between the MoS_2_ and WS_2_ states. In principle, all of the parameters in equation (2) can be calculated with first principles methods, however, as the 2 × 2 model Hamiltonian is significantly reduced from the full Hamiltonian of the actual system, several parameters are obtained by fitting to the TDDFT-MD results. The good agreement between the model Hamiltonian with the TDDFT-MD results using fitted values for *d*, *ɛ* and *E*_0_ establishes the validity of the approach, as shown in Fig. 2d. Notably, instead of a sinusoidal oscillation of *ρ*_h_, the model correctly predicts the sharp peaks and broad mesas observed in the TDDFT-MD simulations. The ability for such a simple model to capture the nonlinear behaviour present in the TDDFT-MD results and the closeness of the fit parameters to those calculated via first principles (Table 1) validate the physical arguments used to derive it.

**Figure 5:**
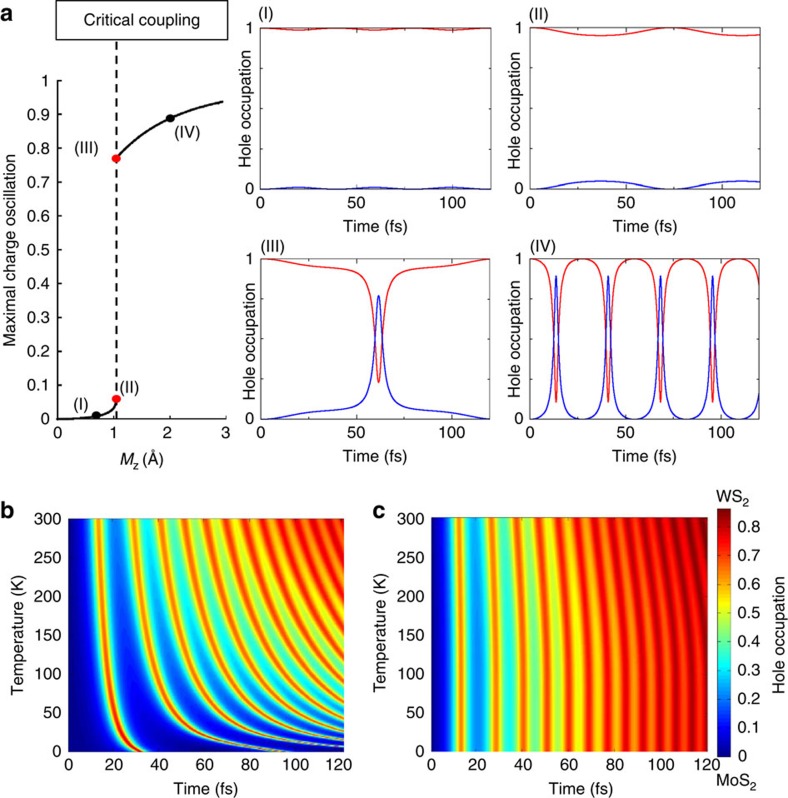
Dynamics of charge transfer. (**a**) Depicts the critical nature of the charge oscillations predicted from the model as a function of the magnitude of the z-component of the dipole transition matrix element, *M*_z_. At the critical point (approximately 1.05 Å, indicated by the dashed line), the dynamics change discontinuously wherein the maximal hole occupation of WS_2_ changes from approximately 0.05 to 0.75. The discontinuous nature of the dynamics with respect to *M*_z_ are apparent by examining the time evolution of *ρ*_h_, shown in the panels, for values of *M*_z_ at the red points lying just to the left (II) and the right (III) of the criticality. (**b**) Colour map indicating the degree of charge transfer as a function of time and temperature calculated from the model interacting with a heat bath containing a receiving mode at 400 cm^−1^. The interaction parameter was determined via a fit to the TDDFT-MD results. (**c**) Colour map indicating the degree of charge transfer as a function of time and temperature, where we have gone beyond the classical picture of ions from the TDDFT-MD simulation by assigning the receiving normal mode energy corresponding to the Bose-Einstein distribution. By directly comparing **b** and **c**, we see that only as we approach room temperature do the classical and quantum pictures of ionic motion yield correspondence.

Investigating how the model describes the physics of charge transfer as a function of *M*_z_, we find that criticality exists and that the hole dynamics undergoes a qualitative change depending on the magnitude of the dipole-induced coupling. Keeping the initial electric field associated with the excitation fixed at 6.9 mV Å^−1^, the maximum amount of dynamic hole transfer as a function of the magnitude of the dipole coupling *M*_z_ is shown in Fig. 5a. For small coupling, this serves to mix the two states leading to small sinusoidal oscillations associated with being in a linear superposition of states. However, after the coupling reaches a critical value, at approximately 1.05  Å, the magnitude of this oscillation changes discontinuously from below 5% to above 75% of the hole population. The emergence of this discontinuity is a result of the inherent non-linearity of the Hamiltonian. Through numerical investigation, we find that the effective potential that governs the dynamics of *ρ*_h_ is bimodal. For small values of *M*_z_, the system does not have enough energy to overcome the barrier separating the two wells and the system dynamics are trapped, yielding only small values of *ρ*_h_ and minimal charge transfer. As *M*_z_ is increased, however, the barrier separating the two wells decreases, until the critical value in which the system dynamics can overcome the barrier leading to much larger oscillations in *ρ*_h_. Physically, beyond this critical value, despite the initial small coupling, the collective behaviour of the holes in the system leads to feedback so that the degree of hole transfer is enough to steadily increase the coupling leading to even more charge transfer. This critical dependence on *M*_z_ highlights the importance of the interlayer spacing (and vdW interaction) on the ultrafast charge-transfer mechanism. Note that the existence of substantial dipole coupling, *M*_z_=〈MoS_2_|*z*|WS_2_〉, relies on the spatial overlap of the |MoS_2_〉 and |WS_2_〉 wavefunctions. Hence, the nature and strength of vdW interaction at the interface, which determines the distance between layers and hence the spatial overlap of the wavefunctions, becomes an essential part of the rapid charge transfer. Furthermore, as electron–phonon coupling is proportional to the magnitude of the dipole oscillation, the fast energy dissipation to phonons and ultimately the ultrafast charge transfer can be viewed as a direct result of this marked charge oscillation resulting from the prerequisite critical dipole coupling of the heterostructure.

**Figure 6:**
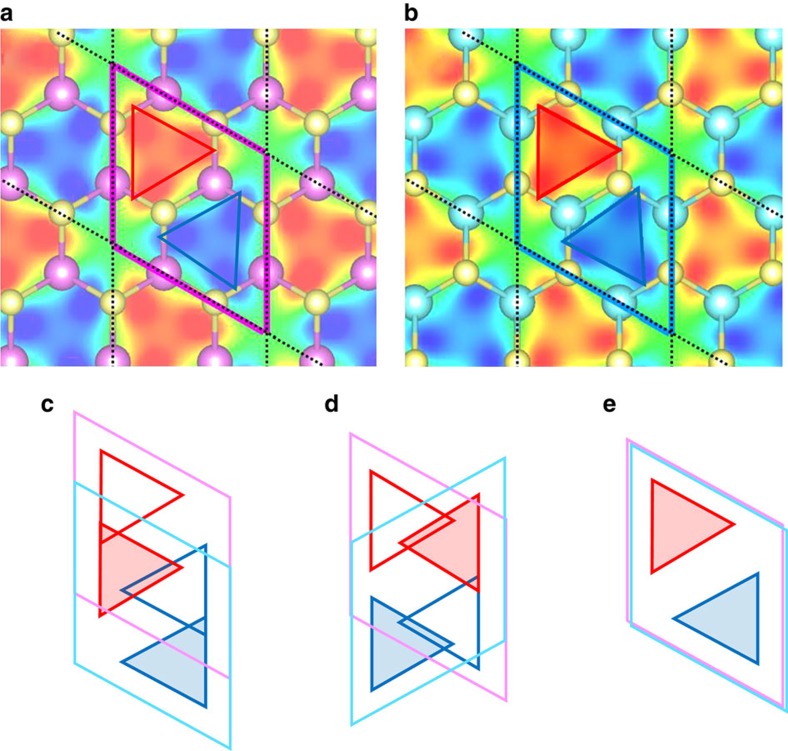
The effect of interface stacking on the electronic coupling. Plots of the wave function associated with the VBM in the vicinity of K for isolated layers of (**a**) |Mos_2_〉 and (**b**) |WS_2_〉. The plots correspond to the values of the wave function above (below) MoS_2_ (WS_2_) at what would be the interfacial plane of a combined heterostructure. Regions where the wavefunctions have a large magnitude have been highlighted by red (blue) triangles to indicate their positive(negative) values. Schematic representations of the wave function alignment between layers for the case of AB', 3R, and 2H stackings are shown in (**c**–**e**), respectively. For the different cases, the unit cells are indicated and the wavefunctions of MoS_2_ (WS_2_) are schematically shown by the open (shaded) triangles. For the 2H stacking case, the wavefunctions line up with each other, yielding maximal spatial overlap and relatively large dipole transition matrix elements.

To extend the model to incorporate interaction with ionic motion, we include a mean-field interaction with a heat bath (see Methods section). Fitting the interaction parameter to a single receiving mode at 400 cm^−1^ leads to a qualitative agreement with the finite temperature TDDFT-MD results, as shown in Fig. 2e,f. Furthermore, this fitting allows us to calculate charge dynamics as a continuous function of temperature in Fig. 5b and improve on the TDDFT-MD results by considering the quantum mechanical nature of the ions in Fig. 5c revealing that near-complete charge transfer takes place within 100 fs even as T approaches 0 K. Figure 5b,c and almost the entirety of the results presented herein refer to the case of 2H stacking between MoS_2_ and WS_2_, which we have predicted to exhibit ultrafast hole transfer. In contrast, for the 3R and AB′ stacking configurations, we find that no hole transfer takes place from MoS_2_ to WS_2_ over the entire simulation period of 120 fs. This result is due to the critical role played by the magnitude of the dipole transition matrix element in the onset of the charge oscillations. For both the 3R and AB′ stackings, we find *M*_z_ to be far below this critical threshold, both being approximately 10^−5^ Å. This sensitivity to stacking is also seen in the transfer integral, where the electronic coupling of the 2H case is found to be 132 meV, many orders of magnitude larger than either the 3R or AB′ cases. This sensitivity on the atomistic details of the interface can be traced back to the *C*_3*v*_ symmetry of the wavefunctions associated with the VBM of the isolated layers of |MoS_2_〉 and |WS_2_〉, which are shown in Fig. 6a,b, respectively. Regions of positive and negative wave function are highlighted by the red and blue triangles, respectively. Looking at the case of AB′ stacking, schematically shown in Fig. 6c, it can be seen that due to the shift in the unit cell, there is very little spatial overlap in the wave function of MoS_2_ (represented by the open triangles) and that of WS_2_ (represented by the shaded triangles). This can also be seen in the case of 3R stacking, shown in Fig. 6d, where the small overlap that does exist tends to cancel. Conversely, for 2H stacking shown in Fig. 6e, the wavefunctions line up entirely, yielding maximal spatial overlap and an associated much stronger coupling.

## Discussion

The TDDFT-MD modelling was performed for the most stable 2H stacking. However, we note that experimental studies of the excited-state hole dynamics have been carried out on far less-controlled samples than the perfect stacking since it is based on mechanical pealing and transfer^20^. The low energy of the 2H stacking suggests that despite the nominal random stacking in experiment, either the 2H stacking dominates or the large sample size guarantees that a sufficiently large proportion of isolated regions feature 2H stacking, thereby ensuring fast charge transfer.

The model describing charge transfer in vertical MoS_2_/WS_2_ heterostructure is general and should be applicable to any weakly bound heterostructures where coupling is dominated by the induced electric field. Furthermore, as charge sloshing couples strongly to Γ-point phonons (*A*_1*g*_) associated with modulation of the vdW gap region, ultrafast charge transport across vdW heterostructures may be the rule rather than the exception. As the initiation of this mechanism for transport is critically dependent on the dipole coupling matrix element, this relationship deserves further study. In particular, our findings prompt a number of fundamental questions: What are the root causes of the discontinuity? What are the dynamics at the critical point? As the collective motion of holes is responsible for the criticality, can this mechanism be interpreted as the instability of an excitonic plasma at the interface? While the timescale of the predicted charge oscillations may be difficult to resolve with current experimental techniques, the mechanism for ultrafast charge transport presented here and the critical role of *M*_z_ should be confirmed either by careful preparation of heterostructures with different stacking configurations or through an assortment of vdW heterostructures that feature different intrinsic *M*_z_. Similar to the discontinuity reproduced by the new model, it is expected that vdW heterostructures with intrinsic *M*_z_ coupling larger than *M*_crit_ will exhibit ultrafast charge transport while those with smaller intrinsic coupling, such as 3R and AB′ stacked MoS_2_/WS_2_, will exhibit charge transport on a timescale several order of magnitudes larger.

In summary, a combined *ab initio* TDDFT-MD simulation and analytic modelling reveals the nature of hole transfer as a result of carriers dynamics at the MoS_2_/WS_2_ vdW heterostructures. Despite the weak interlayer vdW binding, the collective motion of the holes is found to lead to strong dynamic coupling due to the transverse electric field associated with charge transfer. In contrast to classical systems, the build-up of an interfacial dipole can serve as an enhancement factor for such an ultrafast transfer in quantum coherent systems. This ultrafast transfer is well described by a simple model Hamiltonian in which, provided the existence of a critical dipole transition matrix element, the collective motion leads to dramatic non-linear charge oscillations that couple strongly to the phonons at the Γ-point. We anticipate that these findings will trigger further fundamental studies and new device concepts over a broad range of two-dimensional layered materials and systems.

## Methods

### Real-time TDDFT-MD

The electron and ion dynamics are simulated within the TDDFT formalism^35^ coupled with MD, within the local density approximation (LDA) as implemented in the SIESTA code^36^,^37^,^38^. We employ Troullier–Martins norm-conserving pseudopotentials and Ceperley–Alder^39^ exchange-correlation functional. Local basis set with double-ζ polarized orbitals is used. The real-space grid is equivalent to a plane-wave cutoff energy of 350 Ry. A 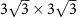 supercell containing 162 atoms with Γ-point for the Brillouin zone integration is used. Note that the K-point of primitive cell is folded to the Γ point in this supercell. In contrast to the well-known underbinding character in the PBE functional, the LDA reproduces the experimental structural parameters quite well for the vdW systems, as shown in Table 2. Although vdW forces are not explicitly included in the LDA formalism, LDA is known to yield a good description of forces between layers in TMDs as indicated by the quantitative agreement of the layer-to-layer vibration modes in these systems, as compared with experimental Raman data^31^,^40^,^41^,^42^,^43^. Furthermore, LDA predicts the preference of the 2H stacking, similar to the vdW density functionals (which are built on top of semi-classical GGA functionals such as PBE)^29^. We consider 3R, AB′ and 2H stacking patterns for the MoS_2_/WS_2_ heterostructures^28^,^29^, but we focus our discussion on the 2H stacking patterns in this study. Testing for the case of 2H stacking indicates that TDDFT-MD calculations using the LDA and PBE+vdW functionals yield quite similar results for the hole transfer dynamics across the heterostructure. We use optimized lattice constants 3.16 Å and all the atoms are fully relaxed until the residual forces are <0.025 eV Å^−1^. In the dynamics calculations, we use NVE ensemble, a time step of 24.19 attoseconds, and the Ehrenfest approximation for ionic motion. Initial ion positions and velocities are obtained from ground-state MD simulations at the target ionic temperature.

### Phonon decomposition

The eigenvectors, 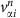, and eigenvalues, *ω*_*n*_, of the normal modes of the system were determined by diagonalizing the dynamical matrix as implemented in SIESTA. Cartesian coordinates of the atomic trajectories during the TDDFT-MD simulation are recast in terms of the normal mode coordinates, *q*_*n*_, by projecting the ionic displacements from equilibrium onto the eigenvectors of the dynamical matrix. In this way, the energy of each normal mode is determined throughout the simulation, according to 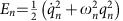.

### Model calculation

The model calculations are performed by numerically integrating the Liouville equation, 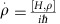, to obtain the density matrix as a function of time. To incorporate the dissipation to the phonon modes observed in the TDDFT-MD calculations at elevated temperatures in the model, the interaction of the system with a heat bath is considered in an average way. In every step in the time evolution of *ρ*, we enforce a Kraus transformation^44^ where


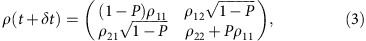


with *P* being the probability that the event of phonon emission with the hole transitioning to a lower energy state (that is, |MoS_2_〉|*n*〉_bath_→|WS_2_〉n+1〉_bath_) takes place in the time δ*t*. Note here that the bath is Markovian and only dissipates energy, we do not consider events which raise the energy of the hole. From Fermi's golden rule, the probability of such a transition is directly proportional to the square of the amplitude of the receiving mode, allowing for the relation of the transition probabilities for different temperatures. Here, however, it is important to note that while the TDDFT-MD calculation is not on the Born–Oppenheimer surface, the ions are still treated as classical objects, and hence the energies of the normal modes in the TDDFT-MD calculations follow a classical equipartition theorem with *E*=*kT*, instead of Bose–Einstein statistics. Hence, for the case of classical ions, assuming a single phonon decay channel associated with a frequency *ω*, we can simultaneously fit both the dynamics at 77 K and 300 K with a single coupling parameter, *g*, where the temperature-dependent transition probability becomes


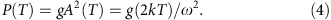


While the amplitude distributions of the normal modes associated with the fully quantum mechanical system are not well represented in the TDDFT-MD, the underlying coupling is well described by the Ehrenfest approximation and hence it is expected that the fitted coupling parameter, *g*, associated with the decay process is still valid. Hence, we can improve the TDDFT-MD simulations to more adequately represent the fully quantum mechanical case by replacing the amplitudes in equation (4) by those correctly determined from the Bose-Einstein distribution yielding,


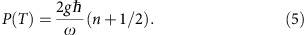


By correcting for this shortcoming, we can go beyond the TDDFT-MD to more accurately calculate charge transfer dynamics at low T, as shown in Fig. 5c. In this case the temperature dependence is much less pronounced and we see nearly complete charge transfer in a 100 fs timescale even as we approach *T*=0 K. This relative insensitivity to temperature is a result of the decay process dominated by high-frequency phonons, which even at room temperature have only very modest phonon occupation (for instance at 300 K, *n*∼0.16). By directly comparing Fig. 5b,c, we see that only as we approach room temperature do the classical and quantum pictures of ionic motion yield correspondence.

## Additional information

**How to cite this article:** Wang, H. *et al*. The role of collective motion in the ultrafast charge transfer in van der Waals heterostructures. *Nat. Commun.* 7:11504 doi: 10.1038/ncomms11504 (2016).

## Figures and Tables

**Table 1:** Values of parameters used in the model Hamiltonian to generate the results presented in Figs 2 and 4 compared to those parameters calculated directly from first-principles calculation.

Parameters	Fitted value	Calculated value
*d* (Å)	4.12	5.49
*E*_0_ (mV^−1^)	6.9	8.4
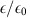	7.6	7.24
*M*_z_ (Å)	1.17	1.17
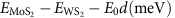	121	121

While the qualitative features of the charge oscillations are well reproduced for the parameter free model, the quantitative agreement between Fig. 2a,d are obtained by using the fitted parameter values in the table above. Note that the values for *M*_z_ and 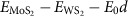 are not fit but instead taken directly from the ground-state wavefunctions and the single particle energies of the TDDFT-MD simulation, respectively. The calculated value of 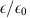 shown is the average of those values obtained from bulk MoS_2_ and bulk WS_2_ of 7.55 and 6.92, respectively. The largest deviation between the fit and calculated values is obtained for the effective distance, 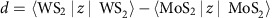, between the slabs. Here we note that the Coulombic attraction between the electron and hole in the dynamics may reduce this distance as observed by the fitted value being 25% shorter than that obtained from the ground-state wavefunctions.

**Table 2:** Experimental and theoretical lattice parameters of MoS_2_ and WS_2_.

	Experiment		Theory	
	MoS_2_	WS_2_	MoS_2_	WS_2_
*a* (Å)	3.160	3.155	3.157	3.157
*c* (Å)	12.294	12.350	12.239	12.360

The experimental results are taken from reference (46). The theoretical results are this work and are calculated within the local density approximation.

## References

[b1] Blankenship R. E. Molecular Mechanisms of Photosynthesis Wiley (2002).

[b2] Kudo A. & Miseki Y. Heterogeneous photocatalyst materials for water splitting. Chem. Soc. Rev. 38, 253–278 (2009). 19088977 10.1039/b800489g

[b3] Alferov Z. I. Nobel Lecture: the double heterostructure concept and its applications in physics, electronics, and technology. Rev. Mod. Phys. 73, 767–782 (2001). 10.1002/1439-7641(20010917)2:8/9<500::AID-CPHC500>3.0.CO;2-X 23686987

[b4] Heeger A. J. 25th anniversary article: bulk heterojunction solar cells: understanding the mechanism of operation. Adv. Mater. 26, 10–27 (2014). 24311015 10.1002/adma.201304373

[b5] Mimura T. The early history of the high electron mobility transistor (HEMT). IEEE Trans. Microw. Theory Tech. 50, 780–782 (2002).

[b6] Deibel C., Strobel T. & Dyakonov V. Role of the charge transfer state in organic donor–acceptor solar cells. Adv. Mater. 22, 4097–4111 (2010). 20803527 10.1002/adma.201000376

[b7] Shaheen S. E. . Energy and charge transfer in organic light-emitting diodes: a soluble quinacridone study. J. Appl. Phys. 85, 7939–7945 (1999).

[b8] Geim A. K. & Grigorieva I. V. Van der Waals heterostructures. Nature 499, 419–425 (2013). 23887427 10.1038/nature12385

[b9] Kosmider K. & Fernandez-Rossier J. Electronic properties of the MoS_2_-WS_2_ heterojunction. Phys. Rev. B 87, 75451 (2013).

[b10] Wang H. . Two-dimensional heterostructures: fabrication, characterization, and application. Nanoscale 6, 12250–12272 (2014). 25219598 10.1039/c4nr03435j

[b11] Lotsch B. V. Vertical 2D heterostructures. Annu. Rev. Mater. Res. 45, 85–109 (2015).

[b12] Pop E., Varshney V. & Roy A. K. Thermal properties of graphene: fundamentals and applications. MRS Bull. 37, 1273–1281 (2012).

[b13] Katsnelson M. I., Novoselov K. S. & Geim A. K. Chiral tunnelling and the Klein paradox in graphene. Nat. Phys. 2, 620–625 (2006).

[b14] Rycerz A., Tworzydlo J. & Beenakker C. W. J. Valley filter and valley valve in graphene. Nat. Phys. 3, 172–175 (2007).

[b15] Sie E. J. . Valley-selective optical Stark effect in monolayer WS_2_. Nat. Mater. 14, 290–294 (2015). 25502098 10.1038/nmat4156

[b16] Cao T. . Valley-selective circular dichroism of monolayer molybdenum disulphide. Nat. Commun. 3, 887 (2012). 22673914 10.1038/ncomms1882 PMC3621397

[b17] Bernardi M., Palummo M. & Grossman J. C. Extraordinary sunlight absorption and one nanometer thick photovoltaics using two-dimensional monolayer materials. Nano Lett. 13, 3664–3670 (2013). 23750910 10.1021/nl401544y

[b18] Splendiani A. . Emerging photoluminescence in monolayer MoS_2_. Nano Lett. 10, 1271–1275 (2010). 20229981 10.1021/nl903868w

[b19] Gutierrez H. R. . Extraordinary room-temperature photoluminescence in triangular WS_2_ monolayers. Nano Lett. 13, 3447–3454 (2013). 23194096 10.1021/nl3026357

[b20] Hong X. . Ultrafast charge transfer in atomically thin MoS_2_/WS_2_ heterostructures. Nat. Nanotechnol. 9, 682–686 (2014). 25150718 10.1038/nnano.2014.167

[b21] Gong Y. . Vertical and in-plane heterostructures from WS_2_/MoS_2_ monolayers. Nat. Mater. 13, 1135–1142 (2014). 25262094 10.1038/nmat4091

[b22] Ceballos F., Bellus M. Z., Chiu H.-Y. & Zhao H. Ultrafast charge separation and indirect exciton formation in a MoS_2_–MoSe_2_ van der Waals heterostructure. ACS Nano 8, 12717–12724 (2014). 25402669 10.1021/nn505736z

[b23] Lee C.-H. . Atomically thin p-n junctions with van der Waals heterointerfaces. Nat. Nanotech. 9, 676–681 (2014). 10.1038/nnano.2014.150 25108809

[b24] Yu Y. . Equally efficient interlayer exciton relaxation and improved absorption in epitaxial and nonepitaxial MoS_2_/WS_2_ heterostructures. Nano Lett. 15, 486–491 (2015). 25469768 10.1021/nl5038177

[b25] Marcus R. A. Electron transfer reactions in chemistry: Theory and experiment. Rev. Mod. Phys. 65, 599–610 (1993).

[b26] van Hal P. A., Meskers S. C. J. & Janssen R. A. J. Photoinduced energy and electron transfer in oligo(p-phenylene vinylene)-fullerene dyads. Appl. Phys. A 79, 41–46 (2004).

[b27] Wasielewski M. R. Photoinduced electron transfer in supramolecular systems for artificial photosynthesis. Chem. Rev. 92, 435–461 (1992).

[b28] van der Zande A. M. . Tailoring the electronic structure in bilayer molybdenum disulfide via interlayer twist. Nano Lett. 14, 3869–3875 (2014). 24933687 10.1021/nl501077m

[b29] He J., Hummer K. & Franchini C. Stacking effects on the electronic and optical properties of bilayer transition metal dichalcogenides MoS_2_, MoSe_2_, WS_2_, and WSe_2_. Phys. Rev. B 89, 75409 (2014).

[b30] Komsa H.-P. & Krasheninnikov A. V. Electronic structures and optical properties of realistic transition metal dichalcogenide heterostructures from first principles. Phys. Rev. B 88, 85318 (2013).

[b31] Berkdemir A. . Identification of individual and few layers of WS_2_ using Raman spectroscopy. Sci. Rep. 3, 1755 (2013).

[b32] Lee C. . Anomalous lattice vibrations of single- and few-layer MoS_2_. ACS Nano 4, 2695–2700 (2010). 20392077 10.1021/nn1003937

[b33] Falke S. M. . Coherent ultrafast charge transfer in an organic photovoltaic blend. Science 344, 1001–1005 (2014). 24876491 10.1126/science.1249771

[b34] Andrea Rozzi C. . Quantum coherence controls the charge separation in a prototypical artificial light-harvesting system. Nat. Commun. 4, 1602 (2013). 23511467 10.1038/ncomms2603 PMC3615481

[b35] Runge E. & Gross E. K. U. Density-functional theory for time-dependent systems. Phys. Rev. Lett. 52, 997–1000 (1984).

[b36] Soler J. M. . The SIESTA method for ab initio order-N materials simulation. J. Phys. Condens. Matter 14, 2745–2779 (2002).

[b37] Meng S. & Kaxiras E. Real-time, local basis-set implementation of time-dependent density functional theory for excited state dynamics simulations. J. Chem. Phys. 129, 054110 (2008). 18698891 10.1063/1.2960628

[b38] Sugino O. & Miyamoto Y. Density-functional approach to electron dynamics: Stable simulation under a self-consistent field. Phys. Rev. B 59, 2579–2586 (1999).

[b39] Ceperley D. M. & Alder B. J. Ground State of the Electron Gas by a Stochastic Method. Phys. Rev. Lett. 45, 566–569 (1980).

[b40] Zhao Y. . Interlayer breathing and shear modes in few-trilayer MoS2 and WSe2. Nano Lett. 13, 1007–1015 (2013). 23432683 10.1021/nl304169w

[b41] Puretzky A. A. . Twisted MoSe2 bilayers with variable local stacking and interlayer coupling revealed by low-frequency raman spectroscopy. ACS Nano 10, 2736–2744 (2016). 26762243 10.1021/acsnano.5b07807

[b42] Huang S. . Low-frequency interlayer raman modes to probe interface of twisted bilayer MoS_2_. Nano Lett. 16, 1435–1444 (2016). 26797083 10.1021/acs.nanolett.5b05015

[b43] Puretzky A. A. . Low-frequency raman fingerprints of two-dimensional metal dichalcogenide layer stacking configurations. ACS Nano 9, 6333–6342 (2015). 25965878 10.1021/acsnano.5b01884

[b44] Kraus K., Böhm A., Dollard J. D. & Wootters W. H. States, Effects, and Operations: Fundamental Notions of Quantum Theory: Lectures in Mathematical Physics at the University of Texas at Austin Springer-Verlag (1983).

[b45] Liang L. & Meunier V. First-principles Raman spectra of MoS_2_, WS_2_ and their heterostructures. Nanoscale 6, 5394–5401 (2014). 24710269 10.1039/c3nr06906k

[b46] Wildervanck J. C. & Jellinek F. Preparation and Crystallinity of Molybdenum and Tungsten Sulfides. Zeitschrift für Anorganische und Allgemeine Chemie 328, 309–318 (1964).

